# Some refinements of operator reverse AM-GM mean inequalities

**DOI:** 10.1186/s13660-017-1557-y

**Published:** 2017-11-14

**Authors:** Jianming Xue

**Affiliations:** Oxbridge College, Kunming University of Science and Technology, Kunming, Yunnan 650106 P.R. China

**Keywords:** 47A63, 47A30, operator inequalities, reverse AM-GM means inequalities, positive linear maps

## Abstract

In this paper, we prove the operator inequalities as follows: Let $A,B$ be positive operators on a Hilbert space with $0 < m \le A,B \le M$ and $\sqrt{\frac{M}{m}} \le2.314$. Then for every positive unital linear map Φ,

$$\Phi^{2} \biggl( {\frac{{A + B}}{2}} \biggr) \le\frac{{ ( {M + m} )^{2} }}{{4Mm}} \Phi^{2} ( {A\mathrel{\sharp} B} ) $$

and

$$\Phi^{2} \biggl( {\frac{{A + B}}{2}} \biggr) \le\frac{{ ( {M + m} )^{2} }}{{4Mm}} \bigl( {\Phi ( A )\mathrel{\sharp} \Phi ( B )} \bigr)^{2}. $$

Moreover, we prove Lin’s conjecture when $\sqrt{\frac{M}{m}} \le 2.314$.

## Introduction

Let $\mathcal{B(H)}$ be the $C^{*}$-algebra of all bounded linear operators on a Hilbert space $\mathcal{H}$. Throughout this paper, a capital letter denotes an operator in $\mathcal{B(H)}$, we identify a scalar with the identity operator *I* multiplied by this scalar. We write $A\ge0$ to mean that the operator *A* is positive. *A* is said to be strictly positive (denoted by $A>0$ ) if it is a positive invertible operator. A linear map $\Phi:\mathcal{B(H)} \to\mathcal {B(K)}$ is called positive if $A\ge0$ implies $\Phi(A)\ge0$. It is said to be unital if $\Phi(I)=I$. For $A,B>0$, the geometric mean $A\mathrel{\sharp} B$ is defined by

$$A\mathrel{\sharp} B = A^{\frac{1}{2}} \bigl(A^{ - \frac{1}{2}} BA^{ - \frac {1}{2}} \bigr)^{{\frac{1}{2}} } A^{\frac{1}{2}}. $$

Let $0 < m \le A,B \le M$. Tominaga [1] showed that the following operator reverse AM-GM inequality holds:

1.1 $$ \frac{{A + B}}{2} \le S ( h )A\mathrel{\sharp} B, $$

where $S ( h ) = \frac{{h^{\frac{1}{{h - 1}}} }}{{e\log h^{\frac{1}{{h - 1}}} }}$ is called Specht’s ratio and $h = \frac {M}{m}$. Indeed,

1.2 $$ S ( h ) \le\frac{{ ( {M + m} )^{2} }}{{4Mm}} \le S^{2} ( h ) \quad ( {h \ge1} ) $$

was observed by Lin [2, (3.3)].

Let Φ be a positive linear map and $A,B > 0$. Ando [3] gave the following inequality:

1.3 $$ \Phi ( {A\mathrel{\sharp} B} ) \le\Phi ( A )\mathrel{\sharp} \Phi ( B ). $$

By (1.1), (1.2) and (1.3), it is easy to obtain the following inequalities:

1.4 $$ \Phi \biggl( {\frac{{A + B}}{2}} \biggr) \le\frac{{ ( {M + m} )^{2} }}{{4Mm}}\Phi ( {A\mathrel{\sharp} B} ) $$

and

1.5 $$ \Phi \biggl( {\frac{{A + B}}{2}} \biggr) \le\frac{{ ( {M + m} )^{2} }}{{4Mm}} \bigl( {\Phi ( A )\mathrel{\sharp} \Phi ( B )} \bigr). $$

Lin [2] proved that (1.4) and (1.5) can be squared:

1.6 $$ \Phi^{2} \biggl( {\frac{{A + B}}{2}} \biggr) \le \biggl({\frac{{ ( {M + m} )^{2} }}{{4Mm}}} \biggr)^{2} \Phi^{2} ( {A\mathrel{\sharp} B} ) $$

and

1.7 $$ \Phi^{2} \biggl( {\frac{{A + B}}{2}} \biggr) \le \biggl({\frac{{ ( {M + m} )^{2} }}{{4Mm}}} \biggr)^{2} \bigl( {\Phi ( A )\mathrel{\sharp} \Phi ( B )} \bigr)^{2}. $$

Meanwhile, Lin [2] conjectured that the following inequalities hold:

1.8 $$ \Phi^{2} \biggl( {\frac{{A + B}}{2}} \biggr) \le S^{2} ( h )\Phi ^{2} ( {A\mathrel{\sharp} B} ) $$

and

1.9 $$ \Phi^{2} \biggl( {\frac{{A + B}}{2}} \biggr) \le S^{2} ( h ) \bigl( {\Phi ( A )\mathrel{\sharp} \Phi ( B )} \bigr)^{2}. $$

For more information on operator inequalities, the reader is referred to [4–7].

In this paper, we will present some operator reverse AM-GM inequalities which are refinements of (1.1), (1.6) and (1.7). Furthermore, we will prove (1.8) and (1.9) if the condition number $\sqrt{\frac{M}{m}} $ is not too big.

## Main results

We begin this section with the following lemmas.

### Lemma 1

([8])

*Let* $A,B>0$. *Then the following norm inequality holds*:

2.1 $$ \Vert {AB} \Vert \le\frac{1}{4} \Vert {A+B} \Vert ^{2}. $$

### Lemma 2

([9])

*Let* $A>0$. *Then for every positive unital linear map* Φ,

2.2 $$ \Phi\bigl(A^{-1}\bigr)\ge\Phi^{-1}(A). $$

### Theorem 1

*If* $0 < m \le A,B \le M$ *for some scalars* $m\le M $, *then*

2.3 $$ \frac{{A + B}}{2} \le\frac{{M + m}}{{2\sqrt{Mm} }}A\mathrel{\sharp} B. $$

### Proof

Put $C = A^{ - \frac{1}{2}} BA^{-\frac{1}{2}} $. Since $\frac{m}{M} \le C \le\frac{M}{m}$, it follows that

$$\biggl[ {C^{\frac{1}{2}} - \frac{1}{2} \biggl( {\sqrt{\frac{m}{M}} + \sqrt{\frac{M}{m}} } \biggr)} \biggr]^{2} \le\frac{1}{4} \biggl( {\sqrt {\frac{M}{m}} - \sqrt{\frac{m}{M}} } \biggr)^{2}, $$

and hence

$$C + 1 \le \biggl( {\sqrt{\frac{M}{m}} + \sqrt{\frac{m}{M}} } \biggr)C^{\frac{1}{2}}. $$

This implies

$$B + A \le \biggl( {\sqrt{\frac{M}{m}} + \sqrt{\frac{m}{M}} } \biggr)A\mathrel{\sharp} B. $$

Thus

$$\frac{{A + B}}{2} \le\frac{{M + m}}{{2\sqrt{Mm} }}A\mathrel{\sharp} B. $$

This completes the proof. □

### Remark 1

By (1.2), it is easy to know that (2.3) is tighter than (1.1).

### Theorem 2

*If* $0 < m \le A,B \le M$ *and* $\sqrt{\frac {M}{m}} \le2.314$ *for some scalars* $m\le M $, *then*

2.4 $$ \biggl( {\frac{{A + B}}{2}} \biggr)^{2} \le \biggl( {\frac{{M + m}}{{2\sqrt {Mm} }}} \biggr)^{2} ( {A\mathrel{\sharp} B} )^{2}. $$

### Proof

Inequality (2.4) is equivalent to

2.5 $$ \biggl\Vert {\frac{{A + B}}{2} ( {A\mathrel{\sharp} B} )^{ - 1} } \biggr\Vert \le\frac{{M + m}}{{2\sqrt{Mm} }}. $$

If $0 < m \le A,B \le\frac{{M + m}}{2}$, we have

2.6 $$ A + \frac{{M + m}}{2}mA^{ - 1} \le\frac{{M + m}}{2} + m $$

and

2.7 $$ B + \frac{{M + m}}{2}mB^{ - 1} \le\frac{{M + m}}{2} + m. $$

Compute

$$\begin{aligned} \biggl\Vert {\frac{{A + B}}{2} \frac{{M + m}}{2}m ( {A\mathrel {\sharp} B} )^{ - 1} } \biggr\Vert &\le \frac{1}{4} \biggl\Vert {\frac{{A + B}}{2} + \frac{{M + m}}{2}m ( {A\mathrel{\sharp} B} )^{ - 1} } \biggr\Vert ^{2} \quad\bigl(\mbox{by (2.1)}\bigr) \\ &\le \frac{1}{4} \biggl\Vert {\frac{{A + B}}{2} + \frac{{M + m}}{2}m \frac{{A^{ - 1} + B^{ - 1} }}{2}} \biggr\Vert ^{2} \\ &\le \frac{1}{4} \biggl( {\frac{{M + m}}{2} + m} \biggr)^{2} \quad\bigl(\mbox{by (2.6), (2.7)}\bigr). \end{aligned} $$

That is,

$$\biggl\Vert {\frac{{A + B}}{2} ( {A\mathrel{\sharp} B} )^{ - 1} } \biggr\Vert \le\frac{{ ( {\frac{{M + m}}{2} + m} )^{2} }}{{4 \frac{{M + m}}{2} m}}. $$

Since $1 \le\sqrt{\frac{M}{m}} \le2.314 $, it follows that

2.8 $$ \biggl( {\sqrt{\frac{M}{m}} - 1} \biggr)^{2} \biggl[ { \biggl( {\sqrt{\frac {M}{m}} } \biggr)^{3} - \frac{{2M}}{m} + \sqrt{\frac{M}{m}} - 4} \biggr] \le0. $$

It is easy to know that $\frac{{ ( {\frac{{M + m}}{2} + m} )^{2} }}{{4 \frac{{M + m}}{2} m}} \le\frac{{M + m}}{{2\sqrt{Mm} }}$ is equivalent to (2.8).

Thus,

$$\biggl\Vert {\frac{{A + B}}{2} ( {A\mathrel{\sharp} B} )^{ - 1} } \biggr\Vert \le\frac{{M + m}}{{2\sqrt{Mm} }}. $$

If $\frac{{M + m}}{2} \le A,B \le M$, we have

2.9 $$ A + \frac{{M + m}}{2}MA^{ - 1} \le\frac{{M + m}}{2} + M $$

and

2.10 $$ B + \frac{{M + m}}{2}MB^{ - 1} \le\frac{{M + m}}{2} + M. $$

Similarly, we get

$$\biggl\Vert {\frac{{A + B}}{2} ( {A\mathrel{\sharp} B} )^{ - 1} } \biggr\Vert \le\frac{{ ( {\frac{{M + m}}{2} + M} )^{2} }}{{4 \frac{{M + m}}{2} M}} \le\frac{{ ( {\frac{{M + m}}{2} + m} )^{2} }}{{4 \frac{{M + m}}{2} m}} \le\frac{{M + m}}{{2\sqrt{Mm} }}. $$

If $m \le A \le\frac{{M + m}}{2} \le B \le M $, we have

$$\begin{aligned} \biggl\Vert {\frac{{A + B}}{2} \frac{{M + m}}{2} \sqrt{Mm} ( {A\mathrel{\sharp} B} )^{ - 1} } \biggr\Vert &\le \frac {1}{4} \biggl\Vert {\frac{{A + B}}{2} + \frac{{M + m}}{2}\sqrt{Mm} ( {A\mathrel{\sharp} B} )^{ - 1} } \biggr\Vert ^{2} \quad\bigl(\mbox{by (2.1)}\bigr) \\ &= \frac{1}{4} \biggl\Vert {\frac{{A + B}}{2} + \frac{{M + m}}{2} \bigl[ { \bigl( {mA^{ - 1} } \bigr)\mathrel{\sharp} \bigl({MB^{ - 1} } \bigr)} \bigr]} \biggr\Vert ^{2} \\ &\le \frac{1}{4} \biggl\Vert {\frac{{A + B}}{2} + \frac{{M + m}}{2} \frac{{mA^{ - 1} + MB^{ - 1} }}{2}} \biggr\Vert ^{2} \\ &\le \frac{1}{4} ( {M + m} )^{2} \quad\bigl(\mbox{by (2.6), (2.10)}\bigr). \end{aligned} $$

That is,

$$\biggl\Vert {\frac{{A + B}}{2} ( {A\mathrel{\sharp} B} )^{ - 1} } \biggr\Vert \le\frac{{ ( {M + m} )^{2} }}{{4 \frac{{M + m}}{2} \sqrt{Mm} }} = \frac{{M + m}}{{2\sqrt{Mm} }}. $$

If $m \le B \le\frac{{M + m}}{2} \le A \le M $, similarly, by (2.1), (2.7) and (2.9), we have

$$\biggl\Vert {\frac{{A + B}}{2} ( {A\mathrel{\sharp} B} )^{ - 1} } \biggr\Vert \le\frac{{M + m}}{{2\sqrt{Mm} }}. $$

This completes the proof. □

### Theorem 3

*Let* Φ *be a positive unital linear map*. *If* $0 < m \le A,B \le M$ *and* $\sqrt{\frac{M}{m}} \le2.314$ *for some scalars* $m\le M $, *then*

2.11 $$ \Phi^{2} \biggl( {\frac{{A + B}}{2}} \biggr) \le \frac{{ ( {M + m} )^{2} }}{{4Mm}}\Phi^{2} ( {A\mathrel{\sharp} B} ) $$

*and*

2.12 $$ \Phi^{2} \biggl( {\frac{{A + B}}{2}} \biggr) \le \frac{{ ( {M + m} )^{2} }}{{4Mm}} \bigl( {\Phi ( A )\mathrel{\sharp} \Phi ( B )} \bigr)^{2}. $$

### Proof

Inequality (2.11) is equivalent to

$$\biggl\Vert {\Phi \biggl( {\frac{{A + B}}{2}} \biggr)\Phi^{ - 1} ( {A \mathrel{\sharp} B} )} \biggr\Vert \le\frac{{M + m}}{{2\sqrt {Mm} }}. $$

If $0 < m \le A,B \le\frac{{M + m}}{2}$, compute

$$\begin{aligned} &\biggl\Vert {\Phi \biggl( {\frac{{A + B}}{2}} \biggr)\frac{{M + m}}{2}m\Phi^{ - 1} ( {A\mathrel{\sharp} B} )} \biggr\Vert \\ &\quad \le\frac{1}{4} \biggl\Vert {\Phi \biggl( {\frac{{A + B}}{2}} \biggr) + \frac{{M + m}}{2}m\Phi^{ - 1} ( {A\mathrel{\sharp} B} )} \biggr\Vert ^{2} \quad\bigl(\mbox{by (2.1)}\bigr) \\ &\quad \le \frac{1}{4} \biggl\Vert {\Phi \biggl( {\frac{{A + B}}{2}} \biggr) + \frac{{M + m}}{2}m\Phi \bigl( { ( {A\mathrel{\sharp} B} )^{- 1} } \bigr)} \biggr\Vert ^{2} \quad\bigl(\mbox{by (2.2)}\bigr) \\ &\quad = \frac{1}{4} \biggl\Vert {\Phi \biggl( {\frac{{A + B}}{2} + \frac{{M + m}}{2}m ( {A\mathrel{\sharp} B} )^{ - 1} } \biggr)} \biggr\Vert ^{2} \\ &\quad \le \frac{1}{4} \biggl\Vert {\Phi \biggl( {\frac{{A + B}}{2} + \frac {{M + m}}{2}m\frac{{A^{ - 1} + B^{ - 1} }}{2}} \biggr)} \biggr\Vert ^{2} \\ &\quad \le \frac{1}{4} \biggl( {\frac{{M + m}}{2} + m} \biggr)^{2} \quad\bigl(\mbox{by (2.6), (2.7)}\bigr). \end{aligned} $$

By $1 \le\sqrt{\frac{M}{m}} \le2.314 $ and (2.8), we have

$$\biggl\Vert {\Phi \biggl( {\frac{{A + B}}{2}} \biggr)\Phi^{ - 1} ( {A \mathrel{\sharp} B} )} \biggr\Vert \le\frac{{M + m}}{{2\sqrt {Mm} }}. $$

If $0 < \frac{{M + m}}{2} \le A,B \le M$, similarly, by (2.1), (2.2), (2.8), (2.9), (2.10) and $\frac {{ ( {\frac{{M + m}}{2} + M} )^{2} }}{M} \le\frac{{ ({\frac{{M + m}}{2} + m} )^{2} }}{m}$, we have

$$\biggl\Vert {\Phi \biggl( {\frac{{A + B}}{2}} \biggr)\Phi^{ - 1} ( {A \mathrel{\sharp} B} )} \biggr\Vert \le\frac{{M + m}}{{2\sqrt {Mm} }}. $$

If $m \le A \le\frac{{M + m}}{2} \le B \le M $, we have

$$\begin{aligned} &\biggl\Vert {\Phi \biggl( {\frac{{A + B}}{2}} \biggr)\frac{{M + m}}{2}\sqrt{Mm} \Phi^{ - 1} ( {A\mathrel{\sharp} B} )} \biggr\Vert \\ &\quad \le\frac{1}{4} \biggl\Vert {\Phi \biggl( {\frac{{A + B}}{2}} \biggr) + \frac{{M + m}}{2}\sqrt{Mm} \Phi^{ - 1} ( {A \mathrel{\sharp} B} )} \biggr\Vert ^{2} \quad\bigl(\mbox{by (2.1)}\bigr) \\ &\quad \le \frac{1}{4} \biggl\Vert {\Phi \biggl( {\frac{{A + B}}{2}} \biggr) + \frac{{M + m}}{2}\sqrt{Mm} \Phi \bigl( { ( {A\mathrel{\sharp} B} )^{ - 1} } \bigr)} \biggr\Vert ^{2} \quad\bigl(\mbox{by (2.2)}\bigr) \\ &\quad = \frac{1}{4} \biggl\Vert {\Phi \biggl( {\frac{{A + B}}{2} + \frac{{M + m}}{2}\sqrt{Mm} ( {A\mathrel{\sharp} B} )^{ - 1} } \biggr)} \biggr\Vert ^{2} \\ &\quad \le \frac{1}{4} \biggl\Vert {\Phi \biggl( {\frac{{A + B}}{2} + \frac {{M + m}}{2} \bigl( {mA^{ - 1} \mathrel{\sharp} MB^{ - 1} } \bigr)} \biggr)} \biggr\Vert ^{2} \\ &\quad \le \frac{1}{4} \biggl\Vert {\Phi \biggl( {\frac{{A + B}}{2} + \frac {{M + m}}{2}\frac{{mA^{ - 1} + MB^{ - 1} }}{2}} \biggr)} \biggr\Vert ^{2} \\ &\quad \le \frac{1}{4} ( {M + m} )^{2} \quad\bigl(\mbox{by (2.6), (2.10)}\bigr). \end{aligned} $$

That is,

$$\biggl\Vert {\Phi \biggl( {\frac{{A + B}}{2}} \biggr)\Phi^{ - 1} ( {A \mathrel{\sharp} B} )} \biggr\Vert \le\frac{{M + m}}{{2\sqrt {Mm} }}. $$

If $m \le B \le\frac{{M + m}}{2} \le A \le M $, similarly, by (2.1), (2.2), (2.7), (2.9), we have

$$\biggl\Vert {\Phi \biggl( {\frac{{A + B}}{2}} \biggr)\Phi^{ - 1} ( {A \mathrel{\sharp} B} )} \biggr\Vert \le\frac{{M + m}}{{2\sqrt {Mm} }}. $$

Thus (2.11) holds.

*A* and *B* are replaced by $\Phi ( A )$ and $\Phi (B )$ in (2.4), respectively, we get (2.12).

This completes the proof. □

### Remark 2

Since $0 < m \le M$, then $\frac{{ ( {M + m} )^{2} }}{{4Mm}} \le [ {\frac{{ ( {M + m} )^{2} }}{{4Mm}}} ]^{2} $. Thus (2.11) and (2.12) are refinements of (1.6) and (1.7), respectively, when $\sqrt{\frac{M}{m}} \le2.314$.

By (1.2) and Theorem 3, we know that Lin’s conjecture (1.8) and (1.9) hold when $\sqrt{\frac{M}{m}} \le2.314$.

### Corollary 1

*Let* Φ *be a positive unital linear map*. *If* $0 < m \le A,B \le M$ *and* $\sqrt{\frac{M}{m}} \le2.314$ *for some scalars* $m\le M $, *then*

$$\Phi^{2} \biggl( {\frac{{A + B}}{2}} \biggr) \le S^{2} ( h ) \Phi ^{2} ( {A\mathrel{\sharp} B} ) $$

*and*

$$\Phi^{2} \biggl( {\frac{{A + B}}{2}} \biggr) \le S^{2} ( h ) \bigl( {\Phi ( A )\mathrel{\sharp} \Phi ( B )} \bigr)^{2}, $$

*where* $S ( h ) = \frac{{h^{\frac{1}{{h - 1}}} }}{{e\log h^{\frac{1}{{h - 1}}} }}$, $h = \frac{M}{m}$.
